# The prominent role of the cerebellum in the social learning of the phonological loop in working memory: How language was adaptively built from cerebellar inner speech required during stone-tool making

**DOI:** 10.3934/Neuroscience.2020020

**Published:** 2020-09-16

**Authors:** Larry Vandervert

**Affiliations:** American Nonlinear Systems, 1529 W. Courtland Ave. Spokane, WA 99205, USA

**Keywords:** cerebellar internal models, cerebellar sequence detection, cerebellum, FOXP2, inner speech, language evolution, phonological loop, stone-tool making, working memory

## Abstract

Based on advances in cerebellum research as to its cognitive, social, and language contributions to working memory, the purpose of this article is to describe new support for the prominent involvement of cerebellar internal models in the adaptive selection of language. Within this context it has been proposed that (1) cerebellar internal models of inner speech during stone-tool making accelerated the adaptive evolution of new cause-and-effect sequences of precision stone-tool knapping requirements, and (2) that these evolving cerebellar internal models coded (i.e., learned in corticonuclear microcomplexes) such cause-and-effect sequences as phonological counterparts and, these, when sent to the cerebral cortex, became new phonological working memory. This article describes newer supportive research findings on (1) the cerebellum's role in silent speech in working memory, and (2) recent findings on genetic aspects (FOXP2) of the role of silent speech in language evolution. It is concluded that within overall cerebro-cerebellar evolution, without the evolution of cerebellar coding of stone-tool making sequences of primitive working memory (beginning approximately 1.7 million years ago) language would not have evolved in the subsequent evolution of Homo *sapiens*.

## 1. Introduction

Over three decades ago, Leiner, Leiner and Dow [1],[2] proposed that the human cerebellum was intimately involved in the evolution of language and thought and that this cerebellar involvement occurred at an unconscious level. A brief return to the development of these ideas will provide the background for the purpose of this article. It was in Leiner, Leiner and Dow's 1989 article [2] where they boldly proposed how cerebellar connections with Broca's language area (Brodmann areas 44 and 45) might have led to increased speed and skill in thought:

Cerebellar connections to Broca's area may not only increase the speed and skill of speaking but also confer other benefits on humans. Because Broca's area communicates with other association areas in the cerebral cortex, the cerebellar signals to Broca's area could increase the speed and skill of such intracortical communication. These communications between cortical association areas are said to comprise the language of thought [3]. Therefore, the process of rational thought may be performed with increased speed and skill in the human brain as a consequence of its [evolutionarily] enlarged cerebro-cerebellar connections. (p. 1006)

The cerebellum increased three- to four-fold in size and the last million years, notably in its lateral cognitive (including working memory) areas [1],[4],[5]. Within this context, the speed and skill of vocalization and sub-vocalization to one's self during difficult tasks among early humans would certainly have been adaptively enhanced by the cerebellum. This idea is supported by Marvel, Morgan and Kronemer [6] who argued how modern roles of verbal working memory grew from and in concert with motor functions of the cerebellum.

Within the same early beginnings of the study of the unconscious cognitive and language functions of the cerebellum, Masao Ito, winner of the Gruber Prize in Neuroscience and the Japan Prize, described how, through repetition (practice) all movement and thought processes become optimized and automatic and are executed unconsciously [7]–[10]. In his important contributions-summarizing book, “The Cerebellum: Brain for an Implicit Self” Ito [10] described his position in a succinct, easy-to-understand manner:

“Brain for an Implicit Self,” reflects my current view of the cerebellum. Its role in the adaptive control of movement is performed unconsciously. Even though voluntary movements, such as those needed to ski, skate, or play a piano, and so on, are performed under conscious awareness (of at least some components of the movements), there is no such awareness when these movements become more refined due to their practice. A similar situation prevails for our thoughts. When we think about some topic repeatedly, the thought becomes more and more implicit; that is, it requires less and less conscious effort, as in intuition. This suggests that the cerebellum aids the self in both movement and thought, but covertly, by use of its internal models. (pp. viii-ix)

Ito [7]–[9] defined cerebellar *internal models* as models of movements, cognitive processes, and social cognition which take place *in* the cerebrum, thus the term “internal” models. See also Van Overwalle, Manto, Leggio & Delgado-Garcia [11] for discussions of cerebellar internal models related to social cognition.

The foregoing general framework of language and unconsciously mediated (implicit) cognitive and social internal modeling functions of the cerebellum have been strongly established in subsequent supportive clinical and brain imaging research [12]–[20].

### 1.1. Purpose

Following directly in the vein of the above more recent and broader cerebellum research, Vandervert [21]–[25] provided arguments on the contributions of the cognitive and social cerebellum in the following areas: the origin of mathematics [21], the leaning of culture [22], and the beginnings of language in the phonological loop of working memory during stone-tool evolution [23]–[25]. The purpose of this article is to provide newer evidence and argument that language evolved principally through inner speech (in the *phonological loop* of working memory) modeled in the cerebellum as it evolved reciprocally with the evolution of stone-tool making. The definition of inner speech in this article follows that of Marvel and Desmond [26] who found cerebellum involvement of inner speech in studies of working memory: “Inner speech is broadly defined as internalized, inaudible verbal thought that may or may not reach conscious awareness and may or may not be accompanied by subliminal vocal activity” (p. 43). In addition, here, inner speech supports phonological rehearsal in working memory [27].

It is important to note before moving on to a discussion of the broader background of cerebro-cerebellar coordination related to inner speech and stone-tool making that the emphasis on functions of the cerebellum herein does not necessarily conflict with approaches that focus on function of the cerebral cortex alone, for example, Alderson-Day and Fernyhough [28], Geva and Fernyhough [29] and Stout and Hecht [30]. Rather, the evidence on functions of the cerebellum in this article intends to bring to bear additional brain mechanisms that provide more detailed and more comprehensive explanations for (1) the initial evolution of inner vocalization toward inner speech, and (2) the subsequent, ongoing cerebellar optimization and increased complexity of the neural patterns behind the evolution of language.

### 1.2. Working memory

Baddeley [31] described working memory as a three-component “brain system that provides temporary storage and manipulation for complex cognitive tasks such as language comprehension, learning and reasoning” (abstract). In Baddeley's scheme, working memory's three components included the following: (1) an attention controlling system which serves as a “central executive,” (2) a visual-spatial sketchpad which manipulates an ongoing cognitively-constructed flow of visual-spatial experience, and (3) a phonological loop which rehearses and stores cognitively-constructed speech-based information. A discussion of Baddeley's [32] episodic buffer component of working memory is beyond the scope of the purposes of this article.

### 1.3. Working memory and the cerebellum: The phonological loop

In accordance with Ito's [10] above description of the implicit self, working memory would be learned in the cerebellum through repetition of all cognitive and social processes that occur in the various levels of schooling (throughout life), daily social interactions, and daily interactions with “tools,” which includes all technology, arts, and music (for example, Ito's above example of playing the piano). This thoroughgoing cerebellar modeling of working memory, notably the central executive and the phonological loop, is very strongly confirmed by a wide variety of cerebellum research contexts [4],[11],[26],[33]–[36]. Note: While Van Overwalle et al. [11] refer to autobiographical memory, autobiographical memory is retrieved in and measured as a function of working memory [37].

### 1.4. The evolution of the phonological loop

An overall description of the evolutionary emergence of the phonological loop was described by Baddeley, Gathercole and Papagno's [38]. In brief, they proposed that “the primary purpose for which the phonological loop evolved was to store unfamiliar sound patterns while more permanent memory records are being constructed” (abstract). Following the findings of Ashida, Cerminara, Edwards, Apps and Brooks [33], Castellazzi, Bruno, Toosy, Casiragi, Palesi, Savini et al. [39], Crespi, Read & Hurd [34] and Saeki, Baddeley, Hitch and Saito [40], it is reasonable to suggest that new, repetitious words would be error-corrected and modeled in the cerebellum in relation to existing phonological working memory. These findings provide a direct neurological parallel to Baddeley, Gathercole and Papagno's description of the purpose and operation of the phonological loop for acquisition of new word forms, a scenario that within Vandervert's [23],[41] proposals places the evolutionary origin of the phonological loop as a concomitant to new, fast-paced cerebellar attention shifting [42] *among* and internal modeling *of* new, repetitious movement requirements and counterpart inner vocalizations across the evolution of stone-tool making.

Vandervert [23]–[25],[41] further argued that this evolutionary view is the most likely to have produced linkage of *detailed sequential cause-and-effect coding with syntactical language modeling* functions in the cerebellum and, thereby, the rise of the phonological loop in the working memory of Homo *sapiens*. The resulting cerebellar internal modeling and optimization of activity in the cerebrum thus produced a creative creature (*sapiens*) that was driven and adaptively selected by the repetitious routines of both increasingly complex stone-tool skill building and technique blending (both requiring detailed cause-and-effect coding in microcomplexes in the cerebellum) by the cerebro-cerebellar system [23],[41]. Note: Neural coding in cerebellar internal models is accomplished by cerebellar corticonuclear microcomplexes which during repetitive skill learning correct movement and cognitive errors toward optimization of the skill at hand [7]–[9]. When any form of the term “coding” is used in this article, it refers to that accomplished by such cerebellar microcomplexes.

### 1.5. The complex repetitiveness of stone-tool making and the emergence of the phonological loop

Following the foregoing arguments on the evolution of the phonological loop, Vandervert [23],[24] proposed that due to the required highly repetitive, detailed action and intense social learning of the actions of others, stone-tool and language evolution was predominantly cerebellum-driven. More specifically, he proposed that this repetitive action and social learning occurred within the framework of theory of mind (ToM). Theory of mind refers to one's simulative mentalizing capacity to make inferences about the mental states of others [43]. Vandervert based this argument on Stout and Hecht's [30] detailed analysis of the rigorous, highly repetitive skill development necessary in learning stone-tool making. Their rather detailed description of the process is quoted at length in order to reveal critical aspects of social/cognitive skill development required of the learner:

Knapping is a “reductive” technology involving the sequential detachment of flakes from a stone core using precise ballistic strikes with a handheld hammer (typically stone, bone, or antler) to initiate controlled and predictable fracture [such prediction would *require internal knowledge or mentalizing of cause-and-effect relationships*]. This means that *small errors in strike execution can have catastrophic, unreversible effects* [italics added]. Experiments by Bril and colleagues have shown that fracture prediction and control is a demanding perceptual-motor skill reliably expressed only in expert knappers [44],[45].

The key bottleneck in the social reproduction of knapping is thus the extended practice required to achieve perceptual-motor competence. This requires mastery of relationships, for example between the force and location of the strike and the morphology, positioning, and support of the core [44],[46],[47] [such mastery would *require neural coding of detailed cause-and-effect relationships*], that are not perceptually available to naïve observers and cannot be directly communicated as semantic knowledge. Attempts to implement semantic knowledge of knapping strategies before perceptual motor skill development are ineffective at best [48],[49], and such knowledge decays rapidly along knapping transmission chains when practice time is limited, even if explicit verbal teaching is allowed [50]. For *observational learning* [italic added], the challenge is to translate visual and auditory information of another's actions to appropriate motor commands for one's own body. This may be accomplished by linking the observed behavior with preexisting internal models [authors here are referring to models in the cerebral cortex, not in the cerebellum] of one's own body and actions through associative learning and stimulus generalization [51],[52]....These learning challenges call for an interactive approach that alternates social-learning opportunities (observation, instruction) with motivated individual practice [53], as commonly seen in coaching and apprenticeship practice. (p. 7862–7863)

Vandervert [23],[24] pointed out that, in their overall description of the evolution of stone-tool knapping and the brain, Stout and Hecht [30] concentrated on functions of the cerebral cortex and did not mention the possible roles of cerebellar internal modeling. To round out the discussion of learning stone-tool knapping, these roles could have included the following: (1) the role of *neural coding in internal models in the cerebellum* for cognitive and socially mediated skill development as described by Ito [7]–[9]; Van Overwalle, Manto, Leggio & Delgado-Garcia [11]; Vandervert [22], (2) the role of inner or silent speech in the phonological loop of working memory in such action as described by Alderson-Day and Fernyhough [28], Crespi, Read and Hurd [34], Mariën, Ackermann, Adamaszek, Barwood, Beaton, Desmond, et al. [18], Marvel and Desmond [26] and Marvel, Morgan and Kronemer [6], and (3) the role of the cerebellum in the internal modeling of repetitive silent speech in *difficult* tasks [18],[33],[39],[40]. Recall, neural coding in cerebellar internal models, see (1) above, is accomplished by cerebellar microcomplexes which during repetitive skill learning correct movement and cognitive errors toward optimization of the skill at hand [7]–[9].

### 1.6. A fuller, actual story behind observational learning?

Combing the above three points, it can be argued that the likely story behind the workings of the role of *observational learning* during stone-tool knapping was *not* overt semantic/verbal communication [which Stout and Hecht [30] pointed out was ineffective], but the ancient learner's inner vocalization that coded appropriate knapping skills. That is, since it has been convincingly shown that the cerebellum learns internal models (coding in cerebellar microcomplexes) of the repetitive actions of the body and sounds of other's [11],[54]–[57], and (1) since overt “semantic” instructions do little good in transferring knapping skills, and (2) verbal working memory is modeled in the cerebellum [4],[26],[35], it is reasonable to suggest that the repetitive body movement and sounds that are learned by the learner are coded in cerebellar internal models of inner vocalization or inner speech.

Following the foregoing research, it is suggested that as the learner observes repetitions of the teacher's knapping movements and sounds, the learner rehearses associated sub-vocalizations in working memory, and, thereby, the sub-vocalizations are modeled in the cerebellum. The resulting internal models of sub-vocalization are optimized, de-composed and blended [58],[59],[60] in the adaptive evolution of inner-speech and language. How such cerebellum-driven modeling of inner vocalizations could have led to the evolution of language is described in the next sections.

## 2. The adaptive selection of rapid shifts of attention linking existing visual-spatial working memory with newly articulated inner vocalization

Vandervert [41] proposed the following scenario for the evolution of language in both phylogeny and the development of language in ontogeny:

In phylogeny, new environmental challenges set in motion the decomposition and re-composition of cerebellar internal models [58],[60] related to situation-specific visual spatial moments and of likewise decomposed/re-composed vocalization patterns linked to those situation-specific moments. These new situation-specific visual spatial moments and their linked situation-specific sound patterns were *blended* proportionately to meet the requirements of the new, challenging situation [59]....The blending process would have resulted in the gradual emergence of a working memory where moments representing cause-and-effect relationships could be quickly tagged into long-term memory using sub-vocal or vocal tags and which, subsequently, could be rapidly accessed from long-term memory using, again, sub-vocal or vocal tags to meet a variety of fast-moving environmental situations. (p. 321)

### 2.1. The state of evolving working memory 1.7 million years ago

Within this context, Vandervert [41] argued that the detailed cause-and-effect relationships required in attention-driven cerebellar modeling [42] of stone-tool making led to decomposition and blending toward *new* cerebellar internal models [7],[9],[10],[61],[62] of visual-spatial working memory and movement coupled with both overt and inner vocalizations in working memory. He further described how such internal models are then fed forward to the cerebral cortex where they are experienced in working memory and give rise to action. This state of working memory likely existed in early humans approximately 1.7 million year ago with early intentional stone modification where it is estimated that technology levels became related to brain evolution [30]. Such primitive inner vocalization likely played all of the many different roles in working memory as appear in the modern inner speech of Homo *sapiens*. See Alderson-Day and Fernyhough [27] for excellent discussions of these roles of inner speech. Marvel, Morgan and Kronemer [6] convincingly argued that these modern roles of inner speech in working memory grew from (were adaptively selected from) such primitive roles.

Specifically within this regard, Vandervert [23],[24],[41] suggested that this early stone era was the basis of the adaptive selection among cerebellar internal models (via decomposition and blending as described above) from vocalization toward primitive speech and primitive inner speech. Through this decomposition and blending, cerebellar internal models for sub-vocal speech and primitive inner speech would have adaptively increased the detailed quality of prediction of the effects of stone work. In addition, sub-vocal speech and primitive inner speech rehearsal during stone work would have helped retain constantly *new*, simple cause-and-effect relationships in memory [26], and would have permitted increased mental manipulation, understanding and automaticity of execution of knapping movements commensurate with inner speech coding of sequences of cause-and-effect relationships. Such an adaptive selection of “verbal” material from vocalization in early working memory is supported by Mariën, Ackermann, Adamaszek, Barwood, Beaton, Desmond, et al. [18].

This overall evolutionary scenario is strongly in sync with Baddeley, Gathercole and Papagno's [31] proposal that “the primary purpose for which the phonological loop evolved is to store unfamiliar sound patterns while more permanent memory records are being constructed” (abstract). Following more recent support for this comes from the findings of Castellazzi, Bruno, Toosy, Casiragi, Palesi, Savini et al. [39] and Mariën, Ackermann, Adamaszek, Barwood, Beaton, Desmond, et al. [18], it is reasonable to suggest that new, repetitious words would be error-corrected and modeled in inner speech coding the cerebellum in relation to existing working memory. That is, this newer evidence provides a direct neurological parallel to Baddeley, Gathercole and Papagno's description of the purpose and operation of the phonological loop for acquisition of new word forms, a scenario that within Vandervert's [23]–[25],[41] proposals places the evolutionary origin of the phonological loop as a concomitant to the rapid attention shifting and inner speech required in the evolution of stone-tool making. Moreover, Vandervert's proposal is supported by Crespi, Read and Hurd's [34] recent findings on roles of the FOXP2 gene in language evolution:

To the extent that the adaptive amino acid evolution of FOXP2, along the human lineage, affected phenotypes comparable to those implicated here, *our results would suggest that inner speech played an important role in the origin and evolution of human language* [italics added]. This is an interesting hypothesis given evidence for causal connections of inner speech with abstract thought, cognitive performance, aspects of learning and development, and default-mode network mental functions [28],[63],[64]). *More generally, partial functional overlaps of outer with inner speech, and inner speech with relational and abstract thought, may have been important in scaffolding the enhanced sophistication of cognition and intelligence that typifies humans compared to other animals that vocalize* [italics added] [28],[65]. (p. 38)

## 3. Conclusions

Through the learning of cause-and-effect *sequencing* of movement and thought, cerebellar internal models [23],[18],[62] make new inner speech sounds (primitive or modern) faster, more consistent and more appropriate (collectively, optimize) toward the task at hand [8],[9]. In stone-tool making (both in ontogeny and phylogeny) this cerebellar optimization of the execution of knapping tasks through new inner-speech cause-and-effect sequencing provided the critical adaptive advantage toward the emergence of language and of Homo *sapiens*. Following Vandervert [23],[24], it is suggested that without this internal modeling of phonological sequencing by the evolving cerebellum, the sequence manipulation inherent in language-driven thought [as theorized by Leiner, Leiner and Dow [2] at the beginning of this article] could not have evolved in the cerebral cortex.

Within the foregoing research context, newer supportive research findings strongly support the cerebellum's prominent role in silent speech in the evolution of phonological working memory. Moreover, recent findings on the role of the FOXP2 gene in language production support the role of inner speech in the evolution of language, a major contention of Vandervert [23]–[25].
